# Dietary fermentable polyols fuel gut inflammation through M1 macrophage polarization and gut microbiota

**DOI:** 10.1016/j.isci.2025.112934

**Published:** 2025-06-19

**Authors:** Kensuke Sato, Miwa Tomioka, Masahiro Akiyama, Yasuyuki Matsuda, Hideki Hara, Haruki Sasa, Yosuke Kurashima, Joe Inoue, Shinji Fukuda, Yun-Gi Kim

**Affiliations:** 1 Department of Microbiology, School of Pharmacy, Kitasato University, Tokyo 108-8641, Japan; 2 Research Center for Drug Discovery, Faculty of Pharmacy and Graduate School of Pharmaceutical Sciences, Keio University, Tokyo 105-8512, Japan; 3 Institute for Advanced Biosciences, Keio University, Yamagata 997-0052, Japan; 4 Graduate School of Media and Governance, Keio University, Kanagawa 252-0882, Japan; 5 Department of Infectious Diseases, Division of Microbiology and Immunochemistry, Asahikawa Medical University, Hokkaido 078-8510, Japan; 6 Department of Innovative Medicine, Graduate School of Medicine, Chiba University, Chiba 260-8670, Japan; 7 Institute for Advanced Academic Research, Chiba University, Chiba 260-8670, Japan; 8 Chiba University Synergy Institute for Futuristic Mucosal Vaccine Research and Development, Chiba 260-8670, Japan; 9 Research Institute of Disaster Medicine, Chiba University, Chiba 260-8670, Japan; 10 Chiba University-University of California, San Diego Center for Mucosal Immunology, Allergy and Vaccine (CU-UCSD cMAV), Department of Medicine, School of Medicine, San Diego, CA 92093-0063, USA; 11 Division of Biochemistry, Faculty of Pharmacy and Graduate School of Pharmaceutical Sciences, Keio University, Tokyo 105-8512, Japan; 12 Transborder Medical Research Center, University of Tsukuba, Ibaraki 305-8575, Japan; 13 Gut Environmental Design Group, Kanagawa Institute of Industrial Science and Technology, Kanagawa 210-0821, Japan; 14 Laboratory for Regenerative Microbiology, Juntendo University Graduate School of Medicine, Tokyo 113-8421, Japan

**Keywords:** Health sciences, Microbiome, Molecular microbiology, Clinical microbiology

## Abstract

While fermentable oligo- and di-, mono-saccharides and polyols (FODMAPs) have been implicated in exacerbating inflammatory bowel disease (IBD) symptoms, the exact influence of FODMAPs on gut microbiota and inflammation is unclear. Here, we show that sorbitol, a polyol, exacerbates colitis in mice induced by dextran sodium sulfate (DSS). Sorbitol increases the expression of inflammatory genes, including *Il1b,* in the colon, associated with M1 macrophage-related genes elevated in IBD patients. Indeed, sorbitol treatment leads to a higher proportion of M1 macrophages in the colon, worsening colitis, which is reversed in interleukin-1β (IL-1β)-deficient mice and mitigated with antibiotic treatment. Sorbitol alters the composition of gut microbiota and metabolites, with Prevotellaceae and tryptamine positively correlated with colonic M1 macrophages. Tryptamine stimulation enhances M1 macrophage polarization. Taken together, polyol consumption activates intestinal macrophages by altering the gut microbiome, which in turn promotes intestinal inflammation.

## Highlights

• Sorbitol worsens DSS-induced colitis and increases inflammatory gene expression in the colon
• Sorbitol increases IL-1β-producing M1 macrophages and exacerbates inflammation in the colon
• Prevotellaceae and tryptamine correlate with M1 macrophages in sorbitol-altered gut microbiota
• Tryptamine promotes M1 polarization of macrophages and is associated with colonic inflammation

## Graphical abstract

**Figure:**
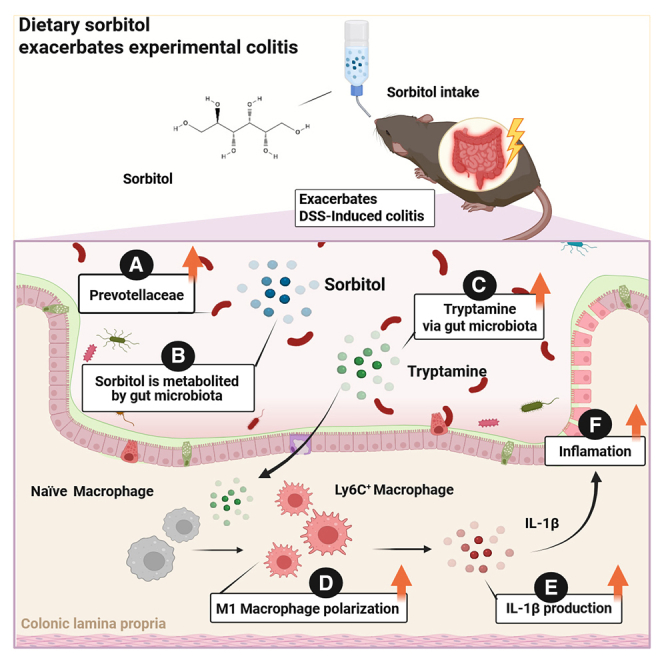

## Introduction

Fermentable Oligo-, Di-, Mono-saccharides, and Polyols (FODMAPs) are poorly absorbed in the gastrointestinal tract, reaching the distal ileum and colon. FODMAPs can be utilized by the gut microbiota and increase fermentation, leading to various associated symptoms, including gas production, abdominal pain, bloating, cramping, distention, and diarrhea.^1^^,^^2^^,^^3^^,^^4^^,^^5^^,^^6^ Mice fed highly fermentable diets exhibit visceral hypersensitivity caused by bacteria-derived histamine and mast cells.^6^ Additionally, dietary fructose aggravates experimental colitis, upregulates inflammatory cytokines in the colon tissue, and increases the total bacteria.^7^ Thus, low-FODMAP diets are recommended to improve the symptoms of inflammatory bowel disease (IBD) patients.^8^^,^^9^

Polyols, especially sorbitol, which is used as a low-calorie sweetener, can also cause polyol intolerance, resulting in diarrhea, due to impaired catabolism by the gut microbiota.^10^^,^^11^^,^^12^^,^^13^ Interestingly, fecal sorbitol concentration in patients with active IBD is significantly higher than that in healthy individuals and those in remission from IBD.^14^ Furthermore, the presence of host sorbitol and its utilization by *Clostridioides difficile* promotes infection in patients with active IBD.^14^^,^^15^

Mucosal immune cells including macrophages maintain gastrointestinal homeostasis by shaping host-microbiota symbiosis.^16^^,^^17^ However, in IBD patients with gut microbiota dysbiosis, macrophages excessively infiltrate the colonic lamina propria and exhibit the M1 inflammatory phenotypes, contributing to intestinal inflammation.^18^^,^^19^^,^^20^ Additionally, the development and progression of IBD are caused by several pro-inflammatory cytokines, such as IL-1β, originating from monocytes and macrophages.^21^

Therefore, dietary polyols may alter gut microbial communities, disrupting immune homeostasis and thereby facilitating gut inflammation. However, the direct impact of dietary sorbitol on the gut microbiome-immune axis and its role in inducing inflammation remain uncertain. Thus, in this study, we aimed to evaluate the effects of sorbitol on gut inflammation. We revealed the effects of dietary sorbitol on gut inflammation in the gut microbiome-immune axis.

## Results

### Dietary polyols exacerbate DSS-induced colitis

**Figure 1:**
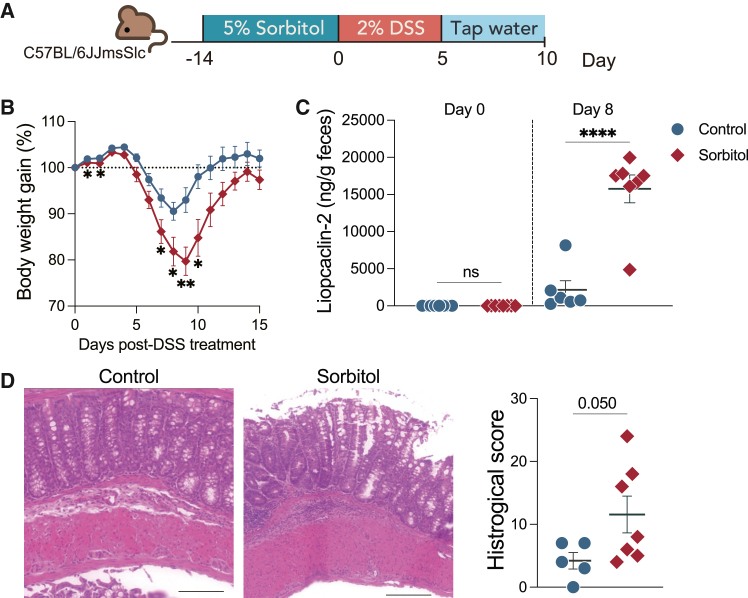
Sorbitol intake exacerbates DSS-induced colitis (A) Diagram illustrating the protocol for sorbitol intake and the DSS treatment (*n* = 6 or 7 samples per group). (B) Body weight gain after DSS treatment compared with day 0 (*n* = 6 or 7 samples per group). (C) Lipocalin-2 in feces on days 0 and 8 post-DSS treatment (*n* = 6 or 8 samples per group). (D) Left: the representative H&E stain section of the murine colon on days 9 post-DSS treatment. Scale bars; 5 μm. Right: the Historical score of colonic tissue injury (*n* = 5 or 7 samples per group). Data are pooled from three independent experiments (B), and plots represent the mean ± SEM. Two-way ANOVA followed by Welch’s t test. ∗∗*p* < 0.01 and ∗*p* < 0.05 (B). Welch’s t test. ∗∗*p* < 0.01 and ∗*p* < 0.05 (C and D).

To examine the effects of dietary sorbitol on experimental colitis, mice were treated with sorbitol for 2 weeks, followed by the administration of dextran sodium sulfate (DSS) (Figure 1A). Sorbitol-treated mice demonstrated a significant loss in body weight compared to the control group (Figure 1B). Consistent with body weight loss, the fecal level of lipocalin-2, an inflammatory marker of colitis, was significantly elevated in sorbitol-treated mice on day 8 post-DSS treatment (Figure 1C). The intake of dietary sorbitol for 2 weeks (on day 0) did not increase the inflammatory marker (Figure 1C). The colon tissue exhibited hypertrophic tunica muscularis externa and inflammatory cell infiltrates of advanced severity (Figures 1D and 1E). Similarly, another polyol, mannitol, was found to exacerbate DSS-induced colitis, as evidenced by increased weight loss and elevated fecal levels of lipocalin-2 (Figures S1A and S1B). These results indicate that dietary polyols promote gut inflammation.

### Sorbitol upregulates the expression of inflammation- and M1 macrophage-related genes in the colon

**Figure 2:**
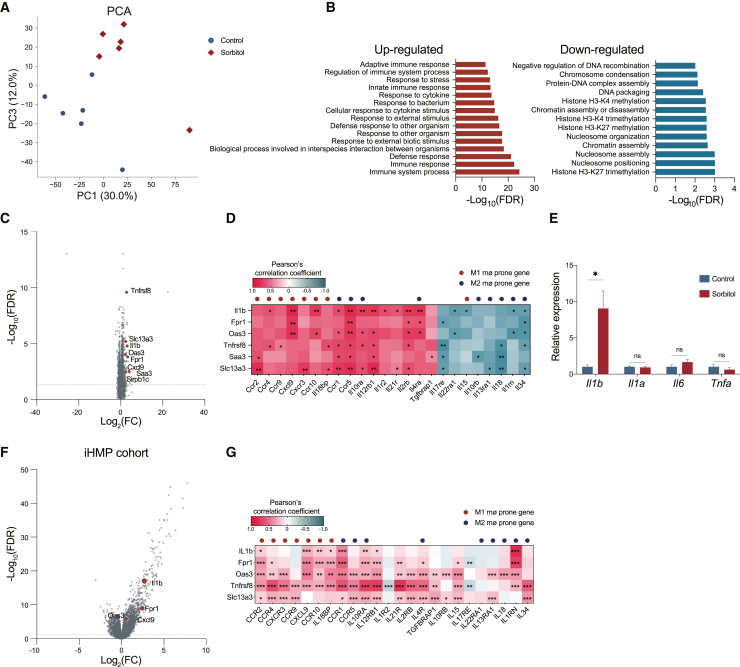
Sorbitol intake upregulates the expression of inflammatory genes in the colon (A) The principal-component analysis (PCA) of gene expression from whole colon RNA sequencing on day 0. Each color and symbol indicates that each group (*n* = 5 or 6 samples per group). (B) Enriched gene ontology (GO) terms based on genes. Right, the significantly upregulated pathways. Left, the significantly down-regulated pathways (*n* = 5 or 6 samples per group). (C) Volcano plot with DEGs. The plots, which are colored red and annotated with gene names, are related to macrophages (*n* = 5 or 6 samples per group). (D) The heatmap with Pearson’s correlation coefficient between the gene expressions related to macrophage and the gene expressions of cytokine or cytokine receptors. The red or blue colored dot indicates the M1 or M2 macrophage-prone genes, respectively (*n* = 5 or 6 samples per group). (E) The relative expression of IL-1β, IL-1α, IL-6, and TNF-α in the colon on day 0 (*n* = 5 or 6 samples per group). (F) Volcano plot with DEGs in Integrative Human Microbiome Project cohort (iHMP) (*n* = 50 samples in non-IBD patients, *n* = 74 samples in IBD patients). The plots, which are colored red and annotated with gene names, are related to macrophages. (G) The heatmap with Pearson’s correlation coefficient between the gene expressions related to macrophage and the gene expressions of cytokine or cytokine receptors (*n* = 50 samples in non-IBD patients, *n* = 74 samples in IBD patients). The red or blue colored dot indicates the M1 or M2 macrophage-prone genes, respectively. Plots represent the mean ± S.E.M. Unpaired t test followed by adjusted *p* value (B, C, and F), Welch’s t test (E), Pearson’s correlation with FDR adjusted *p* value. ∗∗∗*p* < 0.001∗∗*p* < 0.01, and ∗*p* < 0.05 (D and G).

A transcriptome analysis was performed to explore the factors involved in the excessive inflammatory responses induced by sorbitol. The gene expression patterns of the entire colon in the sorbitol group differed from those in the control group (Figure 2A). The most upregulated pathways were related to immune responses in gene ontology terms,^22^ while the nucleosome-related pathways were the most downregulated (Figure 2B). Notably, some of the genes upregulated by sorbitol treatment, *Il1b*, *Fpr1*, *Oas3*, *Tnfrsf8*, *Saa3*, *Slc13a3*, and *Cxcl9*, were highly expressed in macrophages^23^^,^^24^ (Figure 2C). Interestingly, the genes upregulated by sorbitol correlated with cytokines/chemokines or their receptor gene expression. Many positively and negatively correlated genes were expressed in the M1 and M2 macrophages, respectively (Figure 2D).^25^^,^^26^^,^^27^^,^^28^^,^^29^^,^^30^ Consistent with the transcriptome analysis, the expression of the inflammatory cytokine *Il1b*, but not *Il1a*, *Il6*, or *Tnf*, in the colon was significantly upregulated (Figure 2E). Furthermore, within the iHMP IBD cohort,^31^ many of the genes expressed at higher levels in sorbitol-treated mice, including *Il1b*, *Cxcl9*, *Fpr1*, and *Oas3*, were also significantly elevated in IBD patients compared to non-IBD patients and positively correlated with M1-prone genes (Figures 2F and 2G). These results indicate that dietary sorbitol intake enhances the expression of genes related to inflammatory responses and M1 macrophages in the intestine.

### Sorbitol intake promotes M1 macrophage polarization and exacerbates colitis in an IL-1β-dependent manner

**Figure 3:**
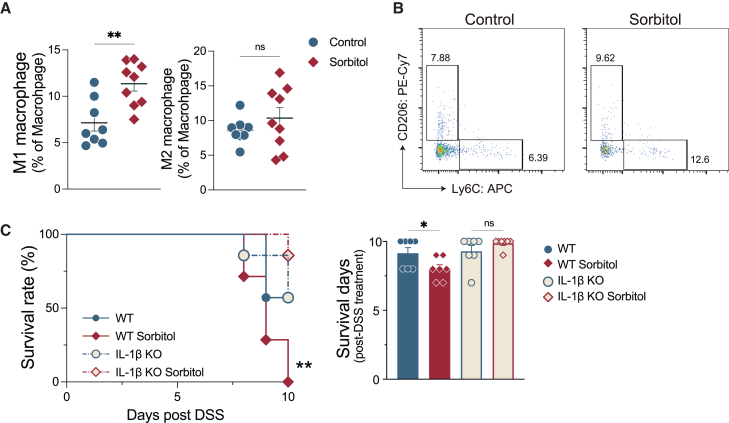
Sorbitol intake promotes M1 macrophage accumulation in the colonic lamina propria and exacerbates colitis in an IL-1β-dependent manner (A) The proportion of M1 or M2 macrophage (Ly6C^+^/CD206^+^, CD45^+^, FVS780^-^, Siglec-F^-^, Ly6G^−^, CD11b^+^, F4/80^+^) in colonic lamina propria (cLP) (*n* = 8 or 9 samples per group). (B) The representative flow cytometry plots of M1 or M2 macrophage (*n* = 8 or 9 samples per group). (C) Right: the survival rate in WT, IL-1β KO mice with or without sorbitol intake after DSS treatment (*n* = 7 samples per group). Left: the survival days post-DSS treatment (*n* = 7 samples per group). Plots represent the mean ± SEM. Welch’s t test (B), log rank test (C), and Sidak’s multiple comparisons tests (C). ∗∗*p* < 0.01 and ∗*p* < 0.05.

Based on these results, we hypothesized that dietary sorbitol increases M1 macrophages in the colon. Consistent with our data, the percentage of M1 macrophages in the colonic lamina propria (cLP) was significantly higher in sorbitol-treated mice, whereas that of M2 macrophages did not change (Figures 3A and 3B). Since sorbitol enhanced the expression of *Il1b*, which is mainly produced from M1 macrophages, we assessed whether the exacerbation of experimental colitis by sorbitol was interleukin-1β (IL-1β) dependent. Unexpectedly, a majority of the mice succumbed to severe colitis when wild-type (WT) mice were cohoused with *Il1b*^−/−^ mice (Figure 3C). Remarkably, sorbitol treatment exacerbated mortality in WT mice following DSS administration, while it had no such effect on *Il1b*^−/−^ mice (Figure 3C). These results suggest that dietary sorbitol increases colonic M1 macrophages and exacerbates colitis in an IL-1β-dependent manner.

### The increase in the colonic M1 macrophages by sorbitol is dependent on gut microbiota

**Figure 4:**
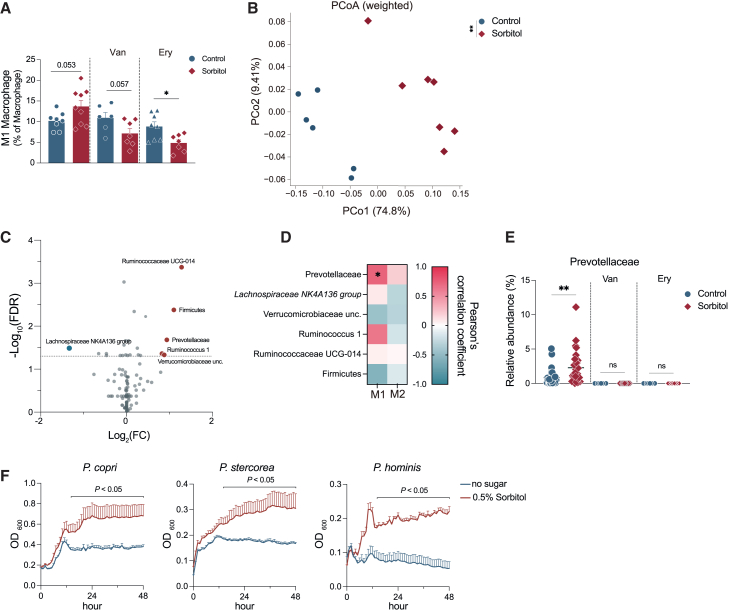
Sorbitol-induced increase in colonic M1 macrophages is dependent on gut microbiota (A) The proportion of M1 macrophages with vancomycin (Van) or erythromycin (Ery) treatment in cLP (*n* = 6–9 samples per group). (B) Principal coordinate analysis (PCoA) plot based on UniFrac distance shows differences in the microbial community. Each symbol and color indicate the time point and group, respectively (*n* = 6 or 7 samples per group). (C) Volcano plot with the differential abundance of gut microbiota in the control versus sorbitol group (*n* = 20 or 21 samples per group). (D) The heatmap with Pearson’s correlation coefficient between the proportion of M1 or M2 macrophages and the relative abundance of gut bacteria (*n* = 6 or 7 samples per group). (E) The relative abundance of Prevotellaceae in murine feces (*n* = 8 or 30 samples per group). (F) The growth of *Prevotella copri* (JCM 13464) (*n* = 12 samples per group), *Prevotella stercorea* (JCM 1346) (*n* = 12 samples per group), and *Prevotella hominis* (JCM 33280) (*n* = 8 samples per group) for 48 h with/without 0.5% sorbitol. Data are pooled from three independent experiments (C, E, and F), and plots represent the mean ± SEM. Welch’s t test (A and E). ∗∗∗*p* < 0.001, ∗∗*p* < 0.01, and ∗*p* < 0.05. Pairwise Permanova, ∗∗*q* value < 0.01 (B). Unpaired t test followed by adjusted *p* value (C), Pearson’s correlation with FDR adjusted *p* value (D), Sidak’s multiple comparisons test (F). ∗∗*p* < 0.01 and ∗*p* < 0.05.

Dietary sorbitol is less absorbed and utilized by the parts of gut microbiota.^10^ Thus, mice were treated with different spectrum of antibiotics^32^ to investigate whether the gut microbiome was involved in the sorbitol-induced increase in M1 macrophage. We have previously shown that treatment with vancomycin and erythromycin depletes both the Bacteroidales and Clostridiales, and Bacteroidales, respectively.^10^^,^^32^ The sorbitol-induced increase in M1 macrophages in cLP was eliminated by antibiotic treatment (Figure 4A). We then assessed the changes in gut microbiota composition after sorbitol intake. The gut microbial community was altered by increasing the relative abundance of several bacterial groups (Figures 4B and 4C). We then performed correlation analysis to examine the relationship between M1 macrophages and gut bacteria. Only the relative abundance of Prevotellaceae showed a statistically significant positive correlation with the percentage of M1 macrophages. While other taxa, including Ruminococcaceae, also showed a positive correlation, the association was not statistically significant (Figure 4D). Additionally, the relative abundance of Prevotellaceae was increased by sorbitol intake, which was abolished by the antibiotic treatment (Figure 4E). To assess whether Prevotellaceae can utilize sorbitol for their growth, three *Prevotella* species (*P. copri*, *P. stercorea*, and *P. hominis*) were grown with sorbitol *in vitro*. Consistent with the *in vivo* results, sorbitol promoted the growth of all three Prevotella species (Figure 4F). These results indicate that sorbitol promotes the polarization of M1 macrophages in a gut microbiota-dependent manner and the growth of *Prevotella* strains.

### Gut microbiota-derived tryptamine promotes the polarization of M1 macrophages

**Figure 5:**
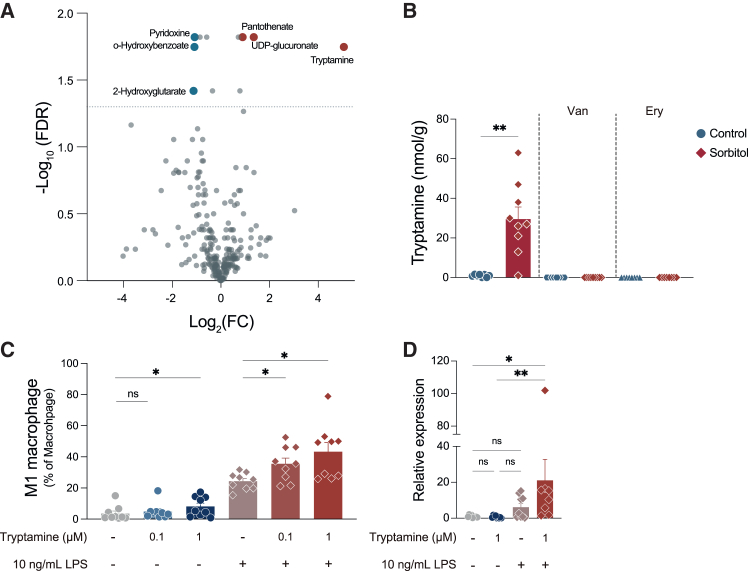
Tryptamine derived from gut bacteria promotes M1 macrophage polarization (A) Volcano plot with the differential abundance of the metabolites in murine cecal content (*n* = 8 or 9 samples per group). (B) The concentration of tryptamine with vancomycin (Van) or erythromycin (Ery) treatment in murine cecal content (*n* = 8 or 9 samples per group). (C) The proportion of M1 macrophage from BMDM stimulated with LPS (10 ng/mL) and different concentrations of tryptamine (*n* = 9 samples per group). (D) The relative expression of IL-1β in BMDM stimulated with/without LPS (10 ng/mL) and 1 μM Tryptamine (*n* = 8 or 9 samples per group). Data are pooled from independent more than three experiments (C and D), and plots represent the mean ± SEM. Unpaired t test followed by adjusted *p* value (A), Welch’s t test (B), Sidak’s multiple comparisons tests (C), Kruskal-Willi’s test (D) ∗∗*p* < 0.01 and ∗*p* < 0.05. ∗∗*q* value < 0.01.

Next, we identified gut-derived metabolites that contributed to the increase in the M1 macrophages by sorbitol. Considering that *Prevotella* is known as a succinate producer^33^^,^^34^^,^^35^^,^^36^^,^^37^^,^^38^^,^^39^ and the gene expression of *Slc13a3,* which is a succinate transporter and promotes the M1 polarization,^40^ was enhanced in murine colon treated with sorbitol (Figure 2C), we suspected gut bacteria-driven succinate may be involved in the polarization of M1 macrophage. Therefore, we first assessed whether sorbitol treatment altered the fecal concentrations of organic acids, including succinate. Consistent with a previous study,^11^ dietary sorbitol elevated the fecal concentrations of short-chain fatty acids (SCFAs), isobutyrate, isovalerate, and valerate but not lactate or succinate (Figure S12A). However, fecal levels of these organic acids did not correlate with the proportion of M1 macrophages in the cLP or the relative abundance of Prevotellaceae (Figure S2B). Then, metabolomics analysis was performed to explore further the metabolites, which revealed that a couple of metabolites were increased by sorbitol administration (Figure 5A). In particular, the intestinal concentration of tryptamine was increased in sorbitol-treated mice, which was abolished by antibiotic treatment (Figure 5B). We then verified whether tryptamine promoted the polarization of M1 macrophages. To reproduce this situation *in vivo*, bone marrow-derived macrophages (BMDMs) were stimulated with tryptamine in the presence or absence of LPS, a major bacterial component. Overnight stimulation with tryptamine tended to increase M2 macrophages rather than M1 macrophages, which was consistent with previous research (Figure S3A).^41^ Long-term stimulation with tryptamine increased the proportion of M1 macrophages in a concentration-dependent manner (Figure 5C). Interestingly, the promotion of M1 macrophage polarization was further enhanced when BMDMs were stimulated with both tryptamine and LPS. In addition, stimulation with both LPS and tryptamine enhanced the expression of IL-1β in BMDMs (Figure 5D). In contrast, treatment with sorbitol or SCFAs, including acetate, butyrate, and propionate, did not promote M1 macrophage polarization in BMDMs after stimulation with LPS (Figures S3B and S3C). Sorbitol did not influence the increase in M1 macrophages by tryptamine (Figure S3C). Taken together, gut microbiota-derived tryptamine, which was increased by sorbitol intake, promoted the polarization of M1 macrophages.

## Discussion

In this study, we revealed a novel pathway within the gut microbiome-immune axis, demonstrating that sorbitol fosters the polarization of M1 macrophages and upregulates the expression of IL-1β through gut bacteria-derived tryptamine. Consequently, this cascade exacerbates experimental colitis. A correlation between the genes upregulated by sorbitol and the M1 macrophage-prone genes was also observed in the iHMP cohort. In patients with active IBD, fecal sorbitol levels were significantly higher compared to both healthy individuals and those in remission from IBD.^4^ Additionally, the aggravation of colitis by sorbitol was dependent on IL-1β. The signaling of IL-1β is a crucial mediator of inflammation and tissue injury in IBD patients.^21^ Several reports indicate that the expression of IL-1β is high in plasma and colonic mucosal tissue^21^^,^^31^^,^^42^^,^^43^ and is associated with disease severity^44^ in IBD patients. In particular, macrophages highly express IL-1β and are the major source of IL-1β in active IBD patients.^43^^,^^45^ Thus, although further clinical validation is needed, dietary intervention may exert beneficial effects in certain IBD patients by suppressing M1 macrophage polarization and associated inflammatory signaling.

In the cohousing experiment with Il1β^−/−^ mice, wild-type mice showed greater susceptibility to DSS-induced colitis. Differences in gut microbiota composition contribute to variability in colitis susceptibility in DSS-treated mice.^46^ Furthermore, the gut microbiota associated with disease susceptibility after DSS treatment could be transferred to other mice.^46^^,^^47^ In our facility, *Il1b*^−/−^ mice may harbor gut microbes that enhance disease severity and outcomes.

Gut microbiota and their metabolites have various effects on the polarization of macrophages. Gut bacteria, such as *Prevotella*, *Akkermansia muciniphila*, and *Proteus mirabilis,* increase macrophages and IL-1β production, exacerbating colitis susceptibility.^40^^,^^46^^,^^47^^,^^48^ Treatment with sorbitol altered the composition of specific microbial groups, increased the relative abundance of Prevotellaceae, and decreased the relative abundance of Lachnospiraceae NK4A136, consistent with a previous report.^40^^,^^46^ Although *in vitro* supplementation with sorbitol significantly promoted the growth of several Prevotella strains, the genes they harbor for sorbitol utilization are still unclear.

The relative abundance of Prevotellaceae was positively correlated with the percentage of M1 macrophages. Intestinal colonization by *Prevotella* exacerbates intestinal inflammation through metabolic changes in microbiota.^46^ Thus, Prevotellaceae may induce inflammatory conditions in the gut by stimulating M1 macrophages. On the other hand, sorbitol intake increased fecal levels of SCFAs. Previous studies have shown that *Ruminococcus* species can directly produce SCFAs, including butyrate.^49^ In contrast, Prevotellaceae do not produce butyrate directly. Instead, their presence may promote butyrate production indirectly through cross-feeding interactions with acetate-utilizing, butyrate-producing bacteria.^46^

SCFAs, including butyrate, suppress M1 macrophage polarization and induce anti-inflammatory responses in IBD.^50^^,^^51^^,^^52^ Consistently, SCFAs did not promote M1 macrophage polarization in BMDMs. These reports and our observations suggest that SCFAs, at least, do not enhance inflammatory responses in macrophages. Further studies are needed to determine the effects of sorbitol on SCFA production and gut inflammation.

Our results indicate that dietary sorbitol enhanced the M1 macrophage population in additional pathways from previous reports.^40^^,^^46^ For instance, *Slc13a3* was upregulated in sorbitol-treated mice. *Slc13a3* is a succinate transporter that promotes the uptake of gut microbiota-derived succinate by macrophages and the transition from a naive macrophage to a proinflammatory state.^40^ The Prevotellaceae are succinate-producing bacteria.^33^^,^^34^^,^^35^^,^^36^^,^^37^^,^^38^^,^^39^^,^^53^ Although there were no differences in succinate concentration in feces between the control and sorbitol groups, upregulation of *Slc13a3* may have resulted from an increase in M1 macrophages.

Exhaustive metabolome analysis revealed that the levels of some metabolites were significantly elevated following sorbitol treatment. In particular, tryptamine markedly increased gut microbiota in a microbiota-dependent manner. Tryptamine, a tryptophan derivative, is synthesized by the gut microbiota and exhibits a high affinity for serotonin type 4 (5-HT4) receptors, eliciting various physiological effects.^54^^,^^55^^,^^56^ Tryptamine also modulates the inflammatory immune response. Stimulation with tryptamine has been shown to reduce the expression of inflammatory cytokines in LPS-stimulated RAW 264.7 cells.^41^^,^^57^ Consistently, overnight stimulation with tryptamine induced the polarization of M2 macrophages in BMDMs while having no significant effect on M1 macrophages.

Prolonged stimulation with tryptamine increased the percentage of M1 macrophages in a concentration-dependent manner, which was further reinforced by the addition of LPS. Additionally, the expression of IL-1β was enhanced by stimulation with tryptamine in the presence of LPS, suggesting that tryptamine induces naive macrophages into a pro-inflammatory state, and additional microbial stimulation can trigger IL-1β production and inflammation. However, there are contradictory reports that bacterial tryptamine attenuates DSS-induced colitis.^58^^,^^59^ Given the limited capacity of tissue-resident macrophages to produce IL-1β,^60^^,^^61^ our *in vitro* experiments, which demonstrated that prolonged stimulation with tryptamine promotes M1 polarization, suggest that tryptamine stimulation may induce the differentiation and polarization of accumulated monocytes to M1 macrophages.

Although evidence for tryptamine production by Prevotellaceae is limited, certain Ruminococcaceae species—such as *Ruminococcus gnavus*—have been reported to promote serotonin biosynthesis and produce tryptamine, which has been associated with diarrhea in IBS patients.^62^ In our data, the relative abundance of Ruminococcaceae was also significantly enriched. Some *Ruminococcus* strains can both produce and metabolize succinate.^53^ This suggests the possibility of complex cross-feeding interactions, in which succinate derived from sorbitol is produced by one taxon and further utilized by another. That is, succinate generated by Prevotellaceae may support tryptamine production by Ruminococcaceae through serotonin biosynthetic pathways. In this context, we focused on Prevotellaceae as a likely upstream contributor that initiates the sorbitol-driven metabolic cascade. This taxon showed both marked enrichment in the sorbitol-supplemented group and a uniquely significant correlation with M1 macrophage frequency, suggesting a central role in shaping the downstream microbial environment. At the same time, the enrichment of Ruminococcaceae indicates that multiple taxa may act synergistically—rather than independently—to contribute to tryptamine accumulation. Therefore, we propose that Prevotellaceae-mediated sorbitol metabolism may provide metabolic substrates such as succinate, which are then utilized by Ruminococcaceae to produce tryptamine. To validate this multi-step model, further investigations are needed, including the isolation and functional characterization of relevant strains and co-culture experiments. Our current findings regarding Prevotellaceae enrichment thus offer a valuable starting point for dissecting the cooperative microbial networks that underlie gut-derived tryptamine production.

Taken together, we found that dietary sorbitol exerts detrimental effects that exacerbate gut inflammation. These results provide evidence that some FODMAPs, including polyols, may adversely affect gut microbiota and intestinal inflammation and support the idea that a low FODMAP diet may be an effective dietary intervention for IBD patients.

### Limitations of the study

There are limitations to this study. While our data suggest tryptamine exacerbates DSS-induced colitis and promotes M1 macrophage polarization, the mechanism by which these phenotypes are potentiated and how sorbitol is linked to tryptamine accumulation and subsequent aggravation of gut inflammation will be important to further decipher. Technical limitations complicate this; however, oral administration of tryptamine does not fully replicate tryptamine production in the colon since tryptamine is absorbed from the small intestine.^63^^,^^64^ Additionally, this study used male mice to avoid potential confounding effects of female sex hormones on immune and inflammatory responses, though future studies incorporating both sexes would provide valuable insights into sex-specific mechanisms and broaden the applicability of these findings. Second, our study was unable to confirm which genes of Prevotellaceae are involved in utilizing sorbitol and whether Prevotellaceae produce tryptamine. To clarify this, isolating Prevotellaceae strains from sorbitol-treated mice is required. Knowing this, it is important to acknowledge the potential for alternative models, including a potential cross-feeding model involving Ruminococcaceae (as discussed previously). Finally, the clinical relevance remains to be determined. Future studies should check for associations between dietary sorbitol and Prevotellaceae abundance in IBD patients.

## Resource availability

### Lead contact

Further information and requests for resources and reagents should be directed to and will be fulfilled by the lead contact, Yun-Gi Kim (kim.yungi@kitasato-u.ac.jp).

### Materials availability

This study did not generate new, unique reagents.

### Data and code availability

• The sequencing data and processed data were deposited into the GEO database (GSE299433). All data reported in this paper will be shared by the lead contact upon request.
• This paper does not report original code.
• Any additional information required to reanalyze the data reported in this paper is available from the lead contact upon request.
• Datasets from the Integrative Human Microbiome Project are available to access at https://www.hmpdacc.org/ihmp/. The accession number for individual datasets is PRJNA398089.

## Acknowledgments

We would like to thank Kyosuke Yakabe, Tomoka Kawashima, Yu Ishiyama, Minami Wakita, Mayu Myobudani, Mitsuko Komatsu, Maho Nakamaru, Noriko Kagata, and Noriko Fukuda for providing technical support, and Tomoya Tsukimi and Kazuki Tanaka for their valuable discussions. This study was supported in part by the JSPS KAKENHI (JP23H02718, JP 23K18223, and JP20H03490 to Y.-G.K.). Graphical abstracts and experimental protocols were created using BioRender.com.

## Author contributions

Y.-G.K. conceived the study and designed the experiments; K.S., M.T., M.A, Y.M, H.H., H.S., Y.K., and S.F. performed the experiments; Y.K., J.I., and S.F provided the resources; K.S., M.T., M.A., Y.M, H.H., and Y.-G.K. analyzed the data; K.S. and Y.-G.K. wrote the manuscript with contributions from all the authors; Y.-G.K. supervised the study.

## Declaration of interests

The authors declare no competing interests.

## STAR★Methods

### Key resources table

**Table:** 

REAGENT or RESOURCE	SOURCE	IDENTIFIER
**Antibodies**		

Purified anti-mouse CD16/32 Antibody (93)	BioLegend	Cat# 101302; RRID: AB_312801
Brilliant Violet 421™ anti-mouse/human CD45R/B220 Antibody	BioLegend	Cat# 103251; RRID: AB_2562905
Purified anti-mouse/human CD45R/B220 Antibody (RA3-6B2)	BioLegend	Cat# RA3-6B2; RRID: AB_312987
Fixable Viability Stain 780	BD Biosciences	Cat# 565388; RRID: AB_2869573
BV510 Rat Anti-Mouse Ly-6G (1A8)	BD Biosciences	Cat# 740157; RRID: AB_2739910
PE, CD11b Monoclonal Antibody (M1/70)	Thermo Fisher Scientific	Cat# 12-0112-83; RRID:AB_273487005
PE-CF594, Rat Anti-Mouse Siglec-F (E50-2440)	BD Biosciences	Cat# 562757; RRID: AB_2687994
PE/Cyanine7, anti-mouse F4/80 Antibody (BM8)	BioLegend	Cat# 123114; RRID: AB_893478
APC, Rat Anti-Mouse Ly-6C (AL-21)	BD Biosciences	Cat# 560595; RRID: AB_1727554

**Bacterial and virus strains**		

*Prevotella copri* (JCM 13464)	Hayashi et al.^38^	N/A
*Prevotella hominis (JCM 33280)*	Liou et al.^39^	N/A
*Prevotella stercorea (JCM 13469)*	Hayashi et al.^38^	N/A

**Chemicals, peptides, and recombinant proteins**		

Liberase™ Research Grade	Roche Diagnostics	Cat# 26628-22-8
Newborn calf serum	Gibco	Cat# 16010-159
Deoxyribonuclease I from bovine pancreas	Sigma-Aldrich	Cat# DN25-5G
Albumin, Bovine Serum, Globulin-free	Nacalai tesque	Cat# 01281-26
Dithiothreitol	Nacalai tesque	Cat# 14128-62
MacConkey Agar Base	Difco™	Cat# 281810
Kanamycin Monosulfate	Nacalai tesque	Cat# 08976-84
TB Green premix Ex Taq (Tli RNaseH Plus)	TaKaRa	Cat# RR420A
Qubit™ dsDNA Quantification Assay Kits	Thermo Fisher	Cat# Q32854
Donkey serum	Sigma-Aldrich	Cat# D9663-10ML
10% SDS Solution	Nippon gene	Cat# 311-90271
ProLong™ Gold Antifade Mountant	Thermo Fisher	Cat# P36930
cOmplete™, Mini Protease Inhibitor Cocktail	Roche	Cat# 11836153001
D(−)-Sorbitol	FUJIFILM Wako Pure Chemical Corporation	Cat# 198-03755
Sodium Dextran Sulfate MW36,000–50,000	MP Biomedicals, LLC	Cat# 593-18795
10% Formalin Solution	Wako Pure Chemical Industries	Cat# 060-03845
AccuDia™ GAM Broth, Modified	Shimadzu Diagnostics Corporation	Cat# 05433
AccuDia™ GAM Semisolid without Dextrose	Shimadzu Diagnostics Corporation	Cat# 302054602
Vitamin K_1_	Tokyo Chemical Industry Co., Ltd.	Cat# P0642
Hemin	Tokyo Chemical Industry Co., Ltd.	Cat# H0008
BD BBL™ Brain Heart Infusion Broth	Difco	Cat# 211059
Advanced Dulbecco’s Modified Eagle Medium/Nutrient Mixture F-12	Gibco	Cat# 12634028
GlutaMAX™ Supplement	Gibco	Cat# 35050061
Recombinant Mouse M-CSF	BioLegend	Cat# 303410-100G
Sodium Acetate	Sigma-Aldrich	Cat# P1880
Sodium Butyrate	Sigma-Aldrich	Cat# S2889-250G
Sodium Propionate	Sigma-Aldrich	Cat# 576404
Lipopolysaccharides from *Escherichia coli* O111:B4	Sigma-Aldrich	Cat# L2630-10MG
Foetal Bovine Serum (FBS), Mexico Origin, Bovine	SERANA	Cat# S-FBS-MX-015
Newborn Calf Serum, heat inactivated, New Zealand origin	Thermo Fisher	Cat# RO-26010074

**Critical commercial assays**		

MagDEA®Dx SV	Precision System Science	Cat# E1300
Mouse Lipocalin-2/NGAL DuoSet ELISA	R&D Systems	Cat# DY1857
RNAlater™ Stabilization Solution	Invitrogen™	Cat# AM7021
PureLink™ RNA Mini Kit	Invitrogen™	Cat# 12183018A

**Deposited data**		

Mouse: C57BL/6JJcl	Sankyo Labo Service Corporation, INC.	N/A
*Il1b* deficient mice	Horai et al.^65^	N/A
Raw and analyzed data (RNA-seq)	This paper	GEO: GSE299433

**Oligonucleotides**		

*Il-1b*; Fwd: GAA ATG CCA CCT TTT GAC AGT G	This study	N/A
*Il-1b*; Rvs: TGG ATG CTC TCA TCA GGA CAG	This study	N/A
*Il-1a* Fwd: AGTATCAGCAACGTCAAGCAA	This study	N/A
*Il-1a* Rvs: TCCAGATCATGGGTTATGGACTG	This study	N/A
*Il-6* Fwd: TGA TGC ACT TGC AGA AAA CA	This study	N/A
*Il-6* Rvs: ACC AGA GGA AAT TTT CAA TAG GC	This study	N/A
*Tnfa* Fwd: CAG GCG GTG CCT ATG TCT C	This study	N/A
*Tnfa* Rvs: CGA TCA CCC CGA AGT TCA GTA G	This study	N/A

**Software and algorithms**		

GraphPad Prism version 10.0.3	GraphPad Software	https://www.graphpad.com/
QIIME2 Version 2022.2.0	QIIME	https://qiime2.org/
Python Version 3.8.3	Python	https://www.python.org/
Matplotlib Version 3.5.0	matplotlib	https://matplotlib.org/
Seaborn Version 0.11.2	seaborn	https://seaborn.pydata.org/#
Flowjo Version 10	FlowJo LLC	https://www.flowjo.com/
Integrative Human Microbiome Project	Lloyd-Price et al.^31^	Accession: PRJNA398089 https://www.hmpdacc.org/ihmp/

**Other**		

Mouse diet: CE-2	CLEA Japan Inc.	N/A
SpectraMax iD3	Molecular Devices	https://www.moleculardevices.co.jp/systems/spectramax-id3-multi-mode-microplate-reader#gref
magLEAD 12gc	Precision system science	https://www.pss.co.jp/product/magtration/lead6-12gc.html
MACSQuant	Miltenyi Biotec	https://www.miltenyibiotec.com/JP-en/products/macs-flow-cytometry/flow-cytometers.html#gref
DLAB DM1424 Hematocrit Centrifuge	DLAB	https://www.dlabsci.com/productDetail?id=61bcdb77ac7dea621e5026d890c90863
Freeze dryer VD-550R	TAITEC	Cat# 0067047-000
StepOnePlus	Thermo Fisher Scientific	https://www.thermofisher.com/order/catalog/product/4376598?SID=srch-srp-4376598
gentleMACS™ Octo Dissociator with Heaters	Miltenyi Biotec	Cat# 130-096-427
gentleMACS™ M Tubes	Miltenyi Biotec	Cat# 130-093-236

### Experimental model and study participant details

#### Mice

Six-week-old, male C57BL/6JJcl mice (Sankyo Labo Service Corporation, INC.) and six-week-old *Il1b*^−/−^ mice^65^ on C57BL/6 background were housed in conventional conditions, with controlled lights (12 h light, 12 h dark cycle), temperature (24 ± 0.5°C), and humidity (40 ± 5%), at the animal facilities of the Faculty of Pharmacy, Keio University (Tokyo, Japan) and Asahikawa Medical University (Hokkaido, Japan). All animal studies were approved by the Institutional Animal Research Committee and Ethics Committee of Keio University and Asahikawa Medical University. Mice had free access to food, CE-2 (CLEA Japan, Inc.), and filter-sterilized drinking water (tap water). After a 5–7-day acclimatization period, the mice were randomly divided into two groups: the control group and the sorbitol group. The mice were rotated between cages to reduce variations in the gut microbiome composition caused by the housing environment. Mice in the sorbitol group were given a solution containing 5% (w/v) sorbitol (FUJIFILM Wako Pure Chemical Corporation) for two weeks. Experimental colitis was induced by administering 2% dextran sodium sulfate (DSS) (MP Biomedicals) dissolved in their tap water for 5 days, after which they were switched back to regular tap water.

### Method details

#### Histological analysis

Whole colons were fixed in a 10% formalin solution (Wako Pure Chemical Industries) and embedded in paraffin. Colon sections were stained with hematoxylin and eosin. Historical injuries were evaluated by September Sapie Co., LTD, based on Grace’s method.^66^

#### Enzyme-linked immunosorbent (ELISA) assay for lipocalin 2 measurement

Feces were suspended in Dulbecco’s phosphate-buffered saline (D-PBS) (−) containing 0.1% Tween 20 to 100 mg/mL and vortexed for 20 min. Following centrifugation at 13,000 ×g at 4°C for 10 min, measurements for lipocalin 2 were conducted using the Mouse Lipocalin-2/NGAL Duo Set (R&D Systems) as per the manufacturer’s instructions.

#### RNA-sequencing analysis

The harvested colon was immersed in RNAlater solution (Invitrogen) and stored at 4°C. For RNA extraction, the colon tissue was processed using PureLink™ RNA mini Kit (Invitrogen)according to the manufacturer’s instructions. RNA sequencing was performed at BGI (Japan). The sequencing data and processed data were deposited into the GEO database (GSE299433).

For primary analysis, data were mapped to murine reference genomes using HITSAT2,^67^ followed by adapter trimming and quality checks assessment using Trim Galore. Gene expression quantification was conducted using StringTie^68^ after converting the alignment results to BAM files using SAM tools.

Secondary analyses, such as differential expression and pathway analyses, were performed using iDEP.^69^

#### Analysis of iHMP transcriptome datasets

Publicly available transcriptome datasets in patients were obtained from the Integrative Human Microbiome Project (iHMP) IBD cohort.^31^ The datasets were processed as described above and mapped to human reference genomes^70^ using STAR.^71^ Differential expression was calculated using PyDEseq2.^72^

#### Reverse transcription-quantitative polymerase chain reaction (RT-qPCR)

The proximal colonic sections were immersed in RNAlater (Thermo Fisher Scientific, Waltham, MA, USA) and stored at −80°C. Total RNA was extracted using the PureLink RNA Mini Kit (Thermo Fisher Scientific) according to the manufacturer’s instructions. The extracted RNA was reverse-transcribed to complementary DNA using ReverTra Ace qPCR RT Master Mix with gDNA Remover (TOYOBO, Osaka, Japan). RT-qPCR was performed using StepOnePlus (Thermo Fisher Scientific) with THUNDERBIRD SYBR qPCR Mix (TOYOBO), wich contains SYBR Green Ⅰ, a DNA-binding fluorescent dye, and the appropriate primer sets (Carlsbad, CA). Relative target messenger RNA levels were calculated with ΔΔCT comparative method. The primer pairs for RT-qPCR were used as follows: *Il-1b*; mIl1b_2_qF (5′-GAA ATG CCA CCT TTT GAC AGT G-3′) and mIl1b_2_qR (5′-TGG ATG CTC TCA TCA GGA CAG-3′), *Il-1a*; mIl1a-qF (5′-AGTATCAGCAACGTCAAGCAA-3′) and mIl1a-qR (5′-TCCAGATCATGGGTTATGGACTG-3′), *Il-6*: mIl6_qF_3 (5′- TGA TGC ACT TGC AGA AAA CA-3′) and mIl6_qR_3 (5′-ACC AGA GGA AAT TTT CAA TAG GC-3′), *Tnfa*: mTnf_qF_2 (5′-CAG GCG GTG CCT ATG TCT C-3′) and mTnf_qR_2 (5′-CGA TCA CCC CGA AGT TCA GTA G-3′).

#### Isolation of cells from the colonic lamina propria (cLP)

Colon sections with the intestinal contents removed were washed twice in D-PBS and separated into intraepithelial cells and other components by incubation in Hank’s Balanced Salt Solution without calcium and magnesium, containing 1 millimolar dithiothreitol and 20 millimolar ethylenediaminetetraacetic acid. Colon sections were cut into tiny fragments, and cells were isolated by incubation in a digestion cocktail containing 0.125 mg/mL DNase I (Merck, Darmstadt, Germany) and 0.2 U/mL Liberase (Roche Diagnostics, Mannheim, Germany), followed by washing with RPMI1640 containing 2% newborn calf serum (NBCS) (Thermo Fisher Scientific, Waltham, MA, USA). Mononuclear cells were isolated via gradient centrifugation using Percoll (GE Healthcare, Chicago, Illinois, US).

#### Flow cytometry

The cells were then stained with various antibodies. For surface and intracellular staining, nonspecific binding was blocked with Fc anti-CD16/32 antibody (1:200) in D-PBS (−) containing 2% NBCS before staining with fluorochrome-conjugated antibodies. The cells were fixed, permeabilized, and stained with antibodies for intracellular staining using the Transcription Factor Buffer Set (BD Biosciences), according to the manufacturer’s instructions. The antibodies used for flow cytometry were as follows; Brilliant Violet 421 anti-mouse CD206 (MMR), PE/Cyanine7 anti-mouse F4/80 (BM8), and FITC anti-mouse/human CD45R/B220 (RA3-6B2) (all from BioLegend), OptiBuild™ BV510 Rat Anti-Mouse Ly-6G (1A8), PE-CF594 Rat Anti-Mouse Siglec-F (E50-2440), and APC Rat Anti-Mouse Ly-6C (all from BD Biosciences), PE CD11b Monoclonal Antibody (M1/70) (Thermo Fisher Scientific). Fixable Viability Stain 780 (BD Biosciences) was used to discriminate dead cells. The stained samples were analyzed using a MACSQuant flow cytometer (Miltenyi Biotech) and FlowJo software (version 10, FlowJo LLC).

#### 16S ribosomal RNA sequencing and analysis

Bacterial DNA was extracted from mouse feces using the E.Z.N.A. Stool DNA Kit Pathogen Detection Protocol (OMEGA) and purified using a magLEAD 12 gc Nucleic Acid Extraction Instrument (Precision System Science Co., Ltd.). DNA was amplified by PCR using primers specific to the V3-V4 regions of the 16S ribosomal RNA gene (key resources table). Sequencing was performed by Cancer Precision Medicine (Japan) on a MiSeq System (Illumina, Inc.). Raw FASTQ files were processed using QIIME2 (Version 2022.2.0)^73^ with denoising using DADA2.^74^ The taxonomy was assigned using the DADA2 implementation of the Ribosomal Database Project classifier in SILVA.^75^^,^^76^

#### Capillary electrophoresis-time of flight mass spectrometry (CE-TOF-MS) for metabolome analysis

The cecum contents were harvested and stored at −80°C until analysis. Subsequently, the cecal contents were lyophilized overnight. Freeze-dried feces (10 mg) were disrupted using 3.0 mm Zirconia Beads (Biomedical Science). The disrupted samples (10 mg) were then homogenized with methanol containing internal standards. After vigorous shaking, distilled water and chloroform were added to the mixture and thoroughly mixed. The supernatant was transferred to a centrifugal filter tube with a 5 kDa limit followed by centrifugation at 4,600 × g and 4°C for 15 min. The filtrate was concentrated by centrifugation at 40°C and reconstituted in distilled water. Ionic metabolites were analyzed using CE-TOF-MS (Agilent Technologies, Japan) in both positive and negative modes. All CE-TOF-MS experiments were performed using an Agilent capillary electrophoresis system for peak annotation and quantification, and the obtained data were processed.

#### Gas chromatography-mass spectrometry for fecal organic acids measurement

The fecal samples were homogenized, weighed, and resuspended in distilled water (w/v). Then, 230 μL supernatant was added with 23 μL of 20% 5-sulfosalicylic acid and 11.5 μL of 1 mM 2-ethyl butyric acid as an internal standard. After centrifugation at 15,000 × g and 4°C for 15 min, 200 μL supernatant was acidified adding with 10 μL of 37% hydrochloric acid, and organic acids were isolated via two rounds of extraction with 1 mL of diethyl ether. Subsequently, 500 μL of organic supernatant was mixed with 50 μL of N-tert-butyldimethylsilyl-N-methyltrifluoracetamide in a new glass vial and incubated at room temperature for 24 h to allow derivatization. To measure the short-chain fatty acids (SCFAs), the derivatized samples were loaded into a chromatography-mass spectrometer (GC-MS; JMS-Q1500GC, JEOL Ltd., Japan) system equipped with an HP-5 capillary column (60 m × 0.25 mm × 0.25 μm, Agilent Technologies, Santa Clara, CA, USA). The concentration of each SCFA was determined by comparing its peak area with that of each standard.

#### The culture of *Prevotella* strains

*Prevotella copri* (JCM 13464),^38^ *Prevotella hominis* (JCM 33280),^39^ and *Prevotella stercorea* (JCM 13469),^38^ which were stored at −80°C, were plated on Brain Heart Infusion broth (Difco) added 1 μL/mL vitamin K1 and hemin (5 μg/mL) and incubated for 5 days at 37°C in anaerobic conditions. After 5 days, colonies were picked and suspended in 4 mL AccuDia™ GAM Broth, Modified medium (mGAM) (Shimadzu Diagnostics Corporation), and incubated overnight at 37°C in anaerobic conditions. Subsequently, the culture medium was centrifuged at 2,000 ×*g* and 4°C for 10 min and washed with D-PBS. The pellets were suspended with 1mL AccuDia™ GAM Semisolid without Dextrose (Shimadzu Diagnostics Corporation) and Aliquots were applied to the medium with/without 0.5% sorbitol. To assess the kinetics of each *Prevotella* strain in the presence of sorbitol, the OD_600_ values were measured every 1 h for 48 h using Spectra Max iD3 (Molecular Devices).

#### Bone marrow-derived macrophages

Bone marrow cells from both the femur and tibia C57BL6/J mice were harvested by flushing with 1% penicillin streptomycin-D-PBS and pipetting up and down into single-cell suspension. After filtration through a 100 μm cell strainer and centrifugation at 500 × *g* for 5 min and 4°C, red blood cells were lysed red blood cells with red blood cell buffer. Followed by centrifuging at 500 × *g* for 5 min and 4°C, bone marrow cells were resuspended with 1% penicillin streptomycin-D-PBS and filtrated through a 100 μm cell strainer. After centrifugation at 500 × *g* for 5 min and 4°C, 5×10^5^/well bone marrow cells were incubated with 2 mL Advanced Dulbecco’s Modified Eagle Medium/Nutrient Mixture F-12 (Gibco) containing 20% fetal bovine serum, 1% penicillin-streptomycin, 1% GlutaMAX (Gibco), and 20 ng/mL recombinant macrophage colony-stimulating factor (BioLegend) for 6 days. To evaluate the polarization of tryptamine to M1 macrophages, cells were stimulated with tryptamine with or without 10 ng/mL lipopolysaccharides (LPS), 1 μM SCFAs including acetate, butyrate, and propionate (Sigma-Aldrich). The medium was changed every three days. After incubation for 6 days, bone marrow-derived macrophages (BMDM) were harvested by adding 0.68 mM ethylenediaminetetraacetic acid, dislodged with a cell scraper, and washed with PBS. Subsequently, BMDMs were centrifuged at 500 × g for 5 min at 4°C, and the pellet was resuspended in Dulbecco’s phosphate-buffered saline without calcium and magnesium (D-PBS (−)) containing 2% newborn calf serum (NBCS). The cells were then stained following the procedure described earlier. To collect RNA, BMDMs were harvested by adding 1mL Sepasol®-RNA I Super G (Nacalai tesque). Subsequently, 200 μL chloroform was added and stirred. After incubating at room temperature for 3 min and centrifuging at 12,000 ×*g* and 4°C for 15 min, 500 μL 2-propanol was added to the collected aqueous phase. After centrifuging at 12,000 ×*g* and 4°C for 10 min, 1 mL of 75% ethanol precipitates were added and centrifuged at 12,000 ×*g* and 4°C for 5 min. The precipitates were dried at room temperature for 5 min and dissolved in water. Harvested RNA was then processed following the procedure described earlier.

#### Statistical analysis

Dunnett’s test was used for statistical analyses of the two groups. Statistical analyses were performed using GraphPad Prism software (version 10.0.3, GraphPad Software Inc.). Statistical significance was determined based on differences with ∗ indicating *p* < 0.05 and ∗∗ indicating *p* < 0.01. Data were visualized using the Python (version 3.8.3), matplotlib (version 3.5.0), and seaborn (version 0.11.2) packages and GraphPad Prism software.
