# Supplementary material for: Dietary fermentable polyols fuel gut inflammation through M1 macrophage polarization and gut microbiota
Source: iScience. 2025 Jun 19;28(7):112934. doi: 10.1016/j.isci.2025.112934 (PMC12270690; doi:10.1016/j.isci.2025.112934)
Supplement: Document S1. Figures S1–S3 [file mmc1.pdf]

iScience, Volume *28*

Supplemental information

Dietary fermentable polyols fuel gut inflammation through M1 macrophage polarization and gut microbiota

Kensuke Sato, Miwa Tomioka, Masahiro Akiyama, Yasuyuki Matsuda, Hideki Hara, Haruki Sasa, Yosuke Kurashima, Joe Inoue, Shinji Fukuda, and Yun-Gi Kim

Supplemental Information

This PDF file includes:

Figures S1 to S3

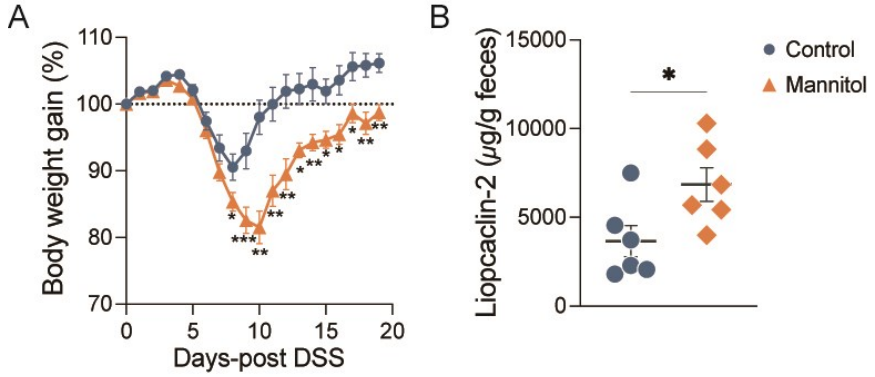

Supplemental Figure 1. Mannitol exacerbates DSS-induced colitis, related to Figure 1.

(A) Body weight gain after DSS treatment compared to day 0. (B) Lipocalin-2 in feces. Two-way ANOVA, followed by Welch's t-test. $^{**}P<0.01$, $^{*}P<0.05$ (A). Welch's t-test (B).

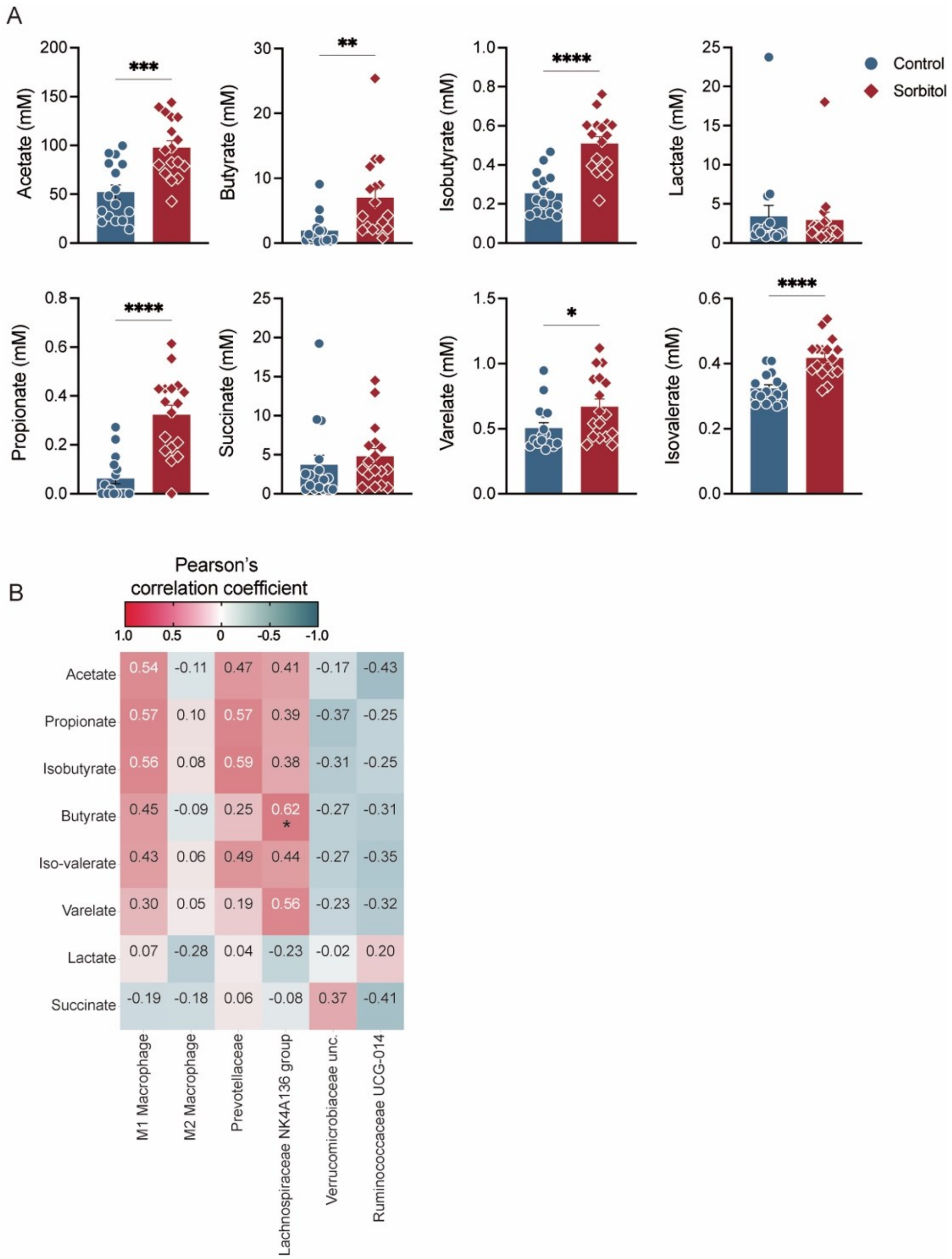

Supplemental Figure 2 Sorbitol intake upregulated the level of organic acids in feces, related to Figure 3.

(A) Levels of organic acids in feces on day 0. (B) Heatmap with Pearson's correlation coefficients between macrophages, gut bacteria, and SCFAs. Data are pooled from independent three experiments, and plots represent the mean and values ± S.E.M. Welch's t-test. ****P<0.0001 ***P<0.001**P<0.01, *P < 0.0. Pearson's correlation with FDR rate-adjusted p-values. ***P<0.001**P<0.01, *P < 0.05 (B).

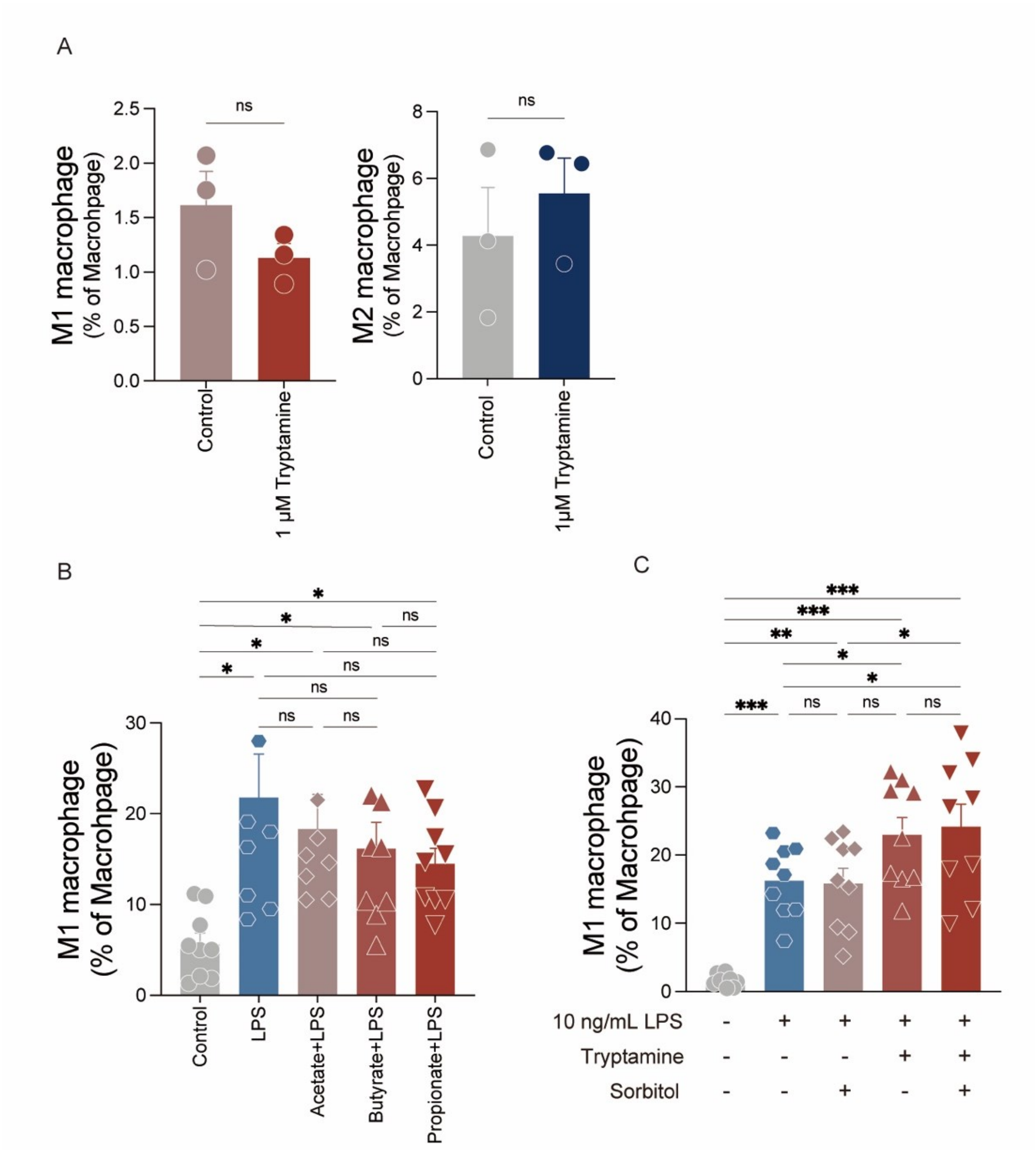

Supplemental Figure 3. Sorbitol, SCFAs, and short-time stimulation with tryptamine did not promote the M1 macrophage polarization, as shown in Figure 5.

(A) Left; The proportion of M1 macrophages in BMDM stimulated with tryptamine. Right; The proportion of M2 macrophages in BMDM stimulated with tryptamine. (B) The proportion of M1 macrophages in BMDM stimulated with LPS (10 ng/mL), acetate, butyrate, and propionate (1 µM). (C) The proportion of M1 macrophages in BMDM stimulated with LPS, tryptamine 1 µM, and sorbitol (3 µM). Data are pooled from three independent experiments (B, C), and plots represent the mean and values ± S.E.M. Paired t-test (A), Tukey multiple comparisons (B, C). ****P* < 0.001, ***P* < 0.01, **P* < 0.05. (A, B).
